# Specific Learning Difficulties: Disparities in Identification

**DOI:** 10.1177/00222194261427006

**Published:** 2026-02-25

**Authors:** Johny Daniel, Julian Elliott, Peter Tymms, Steve Strand

**Affiliations:** 1 Durham University, UK; 2 University of Oxford, UK

**Keywords:** specific learning difficulties, learning disabilities, identification, gender, socioeconomic status, English as an additional language learner, school effects

## Abstract

This study investigates disparities in the identification of specific learning difficulties (SpLD) in England. We estimated multilevel logistic regression models on National Pupil Database records for approximately 540,000 Year 6 students across 14,800 schools. Student-level predictors included academic performance in reading and math, gender, English as an additional language (EAL) status, mobility status, and individual economic deprivation indicator; school-level predictors included average school reading and math attainment, proportion of EAL students, and average deprivation. Substantial between-school variation in SpLD identification was observed. After controlling for attainment and student background characteristics, EAL students had markedly lower odds of identification. Similarly, female students were less likely to be identified for SpLD than their male peers. Furthermore, higher average school-level deprivation predicted reduced identification odds. These findings highlight that systemic and contextual factors, alongside individual learning profiles, shape SpLD identification and raise concerns about equitable access to assessment and support for a diverse group of learners.

Is it appropriate and acceptable for a given child, with a given profile of skills and achievement, to be considered to have a specific learning difficulty (SpLD) if they attend School A rather than School B? While it has long been known that schools as social institutions exert differential effects upon children’s learning (Mortimore et al., 1988; Rutter, 1979), the question posed here is unconcerned with the possible reasons for actual differences between a child’s educational development should they have attended School A rather than School B; neither does it address where one stands in relation to debates over the appropriateness of medical versus social models of disability (Bunbury, 2019; Dwyer, 2022). Rather, it simply asks whether an individual presenting with a particular level of academic achievement would, in one school, be deemed to have an SpLD but be considered not to have such a need in another.

While there is likely to be variation of judgment and procedures between individual schools (Berkeley et al., 2020; Gilmour et al., 2024), any systemic patterns in determining that a given child has, or does not have, a SpLD, reflecting school composition differences of race, gender, religion or social class, would surely be inappropriate. Not only does the SpLD label often provide additional accommodations and resources and, perhaps, more teacher understanding and encouragement (Kashikar et al., 2025), it also often carries a higher status than alternative labels that highlight intellectual or behavioral difficulties (Fish, 2019, 2022). The purpose of this study, therefore, is to investigate whether and how school membership, student-level academic achievement, demographic characteristics, and school-level composition collectively influence variations in the identification of SpLD among upper elementary students in England.

## Identification of SpLD

Following the seminal work of Kirk in the United States (1963), in relation to the category of Learning Disability (LD), the notion of a SpLD being demonstrated by unexpected underachievement was codified in U.S. federal law (U.S. Public Law 115-391) in 2018. In recent years, more countries have adopted this conception (Agrawal et al., 2019; Sideridis, 2007). While the definition remains highly contested (Hajovsky et al., 2025), it delineates the core characteristics of LD/SpLD, which concern unexpected and persistent difficulties in reading, writing, spelling, or mathematical calculation, not principally attributable to sensory, motor, cognitive, emotional, cultural, or economic factors.

In England, the Special Educational Needs and Disabilities (SEND) Code of Practice (Department for Education, 2015) and the Children and Families Act (Department for Education, 2014) form the legislative and statutory framework governing identification, assessment, and support for children and young people with SEND. However, neither of these frameworks provides a specific definition of SpLD. The most referenced definition of a SpLD in England concerns dyslexia, reported in the Rose Report (2009, p. 30) as “*a learning difficulty that primarily affects the skills involved in accurate and fluent word reading and spelling.*” Moreover, a range of English foundations and charities offer varying definitions of SpLD, a divergence that has been linked to inconsistencies in assessors’ identification practices (Daniel et al., 2025).

Although the SEND Code of Practice (Department for Education, 2015) advocates a needs‑led approach where appropriate support should be made available without the necessity of a formal diagnosis, school census data in England indicate that fewer than 2 % of students are recorded with a primary need of SpLD (Daniel & Wang, 2024); this figure is well below international epidemiological estimates of 5% to 10% (e.g., Fletcher et al., 2019). Some estimates for the prevalence of reading difficulties are as high as 1 in 5 individuals (de Jong, 2020; Jimenez et al., 2011). This variation in prevalence and incidence estimates results from the fact that the attributes of SpLD are dimensional rather than categorical (Wagner et al., 2020). This discrepancy suggests that the absence of a shared operational definition, combined with reliance on privately commissioned assessments, may be resulting in a substantial proportion of learners with SpLD remaining unidentified in England. Paradoxically, despite official guidance, formal diagnoses remain widely used in practice as a means of securing additional educational support (Elliott et al., 2024), raising concerns about equitable access to resources.

## Disparities and Contextual Influences in SpLD Identification

### Gender

While recent national data (Daniel & Wang, 2024; Department for Education, 2023) indicate that more boys receive SEND support for identified SpLD (1.12%) than do girls (0.88%), it is unclear whether this reflects meaningful gender differences or identification bias. Supporting the former explanation, international test results, on average, demonstrate that girls score higher in reading skills across multiple countries, education systems, and orthographies, including England, while boys score marginally higher than girls in numeracy skill assessments (Borgonovi et al., 2018). Supporting the latter explanation (i.e., identification bias), Strand and Lindsay (2009) analyzed the National Pupil Database (NPD) and found that girls had significantly lower odds of being identified with SpLD (OR = 0.40). Moreover, their analyses demonstrated that girls were under-represented across all 12 categories of SEND, placing the SpLD disparity within a broader pattern of systemic under-identification of female students for SEND provision.

Furthermore, international data suggest that males are more likely to be identified as having a reading disability, with the ratio of males to females identified ranging from 0.84 to 4.48 across studies, with an average odds ratio of 1.83 in favor of boys (Quinn, 2018). These figures have prompted debate about whether higher identification rates for boys reflect genuine underlying prevalence or systematic bias in referral and assessment. In challenging the “myth” of male vulnerability to reading disability, Shaywitz (1996, p. 98) has argued that a disproportionate number of boys are identified as reading disabled because they are more likely than girls to present with comorbid externalizing disorders, and the hyperactive-impulsive form of Attention-Deficit/Hyperactivity Disorder (Barkley, 2015).

In contrast, girls are more likely to present with internalizing problems (Pennington, 2008). As schools tend to refer to clinical services children with conduct rather than internalizing disorders (Bramlett et al., 2002), disproportionate referral rates of boys and girls for reading-related problems are likely to be an inevitable outcome. While a referral bias of this kind may well be a contributory factor, there nevertheless appear to be meaningful differences between the sexes, given findings from epidemiological studies indicating a male-female ratio of approximately 1.5:1 (Brimo et al., 2021; Flannery et al., 2000; Flynn & Rahbar, 1994). While most studies have not found sex differences in the prevalence of math learning difficulties (Fletcher et al., 2019), some studies have found boys to be over-represented at both ends of the distribution of math performance (Gray et al., 2019; Nowell & Hedges, 1998; Räsänen et al., 2021). This gendered pattern of identification raises questions about the behavioral thresholds that trigger referral, and whether current practices adequately capture the needs of all learners. If identification is influenced as much by behavioral presentation as by academic profile, then current practices may contribute to boys being over-identified and girls under-identified, even where genuine prevalence differences exist.

### English as an Additional Language Learner (EAL) Status

In England, the proportion of students identified as EAL has increased from approximately 7% in 1997 to 20% in 2023 (Lindorff et al., 2025). Several large-scale analyses of the NPD have examined the role of EAL alongside other student characteristics in SEND identification. For example, Strand and Lindsay (2009) used the 2005 NPD census to examine ethnic disproportionality in SEND identification. They found that, compared to White British students, students from most non-White British ethnic backgrounds had significantly lower odds of being identified with SpLD. However, it is important to note that the analysis did not control for students’ academic attainment, which may confound the interpretation of these disparities. Strand and Lindorff (2018) extended earlier analyses by including additional controls to examine whether disproportional representation of student subgroups had changed since 2005. In models that controlled for prior attainment, they found that non-White students had significantly lower odds of identification for SEND categories such as autism and social, emotional, and mental health needs. However, these prior attainment-adjusted models did not include SpLD, and thus, they did not evaluate how achievement and ethnicity jointly influence SpLD identification. Recent work in England (Hutchinson et al., 2025) continues to indicate that EAL students remain around 35% less likely to receive SEND support than monolingual peers, although these findings do not disaggregate students identified with SpLD specifically.

International evidence on the association between EAL status and SEND identification remains mixed. In the U.S., Artiles et al. (2005) found over-identification of EAL students in some contexts, particularly for those with low proficiency in both their first language and English. Whereas Cooc (2018) reported under-identification among Asian-American students, this work examined ethnicity rather than language status and therefore does not directly address EAL identification. In the English context, the practical challenge remains that most dyslexia and other SpLD assessments for EAL students are conducted generally in English, with limited consideration of first-language skills (Daniel et al., 2025), thus creating a risk of both under- and over-identification depending on how language acquisition interacts with assessment practices.

### Socioeconomic Status

Students from socioeconomically disadvantaged backgrounds have been reported to be both over- and under-identified for SEND services. In England, Hutchinson et al. (2025) reported that students from low-income backgrounds were more likely to be identified for SEND services. Campbell (2023) similarly showed that children living in England’s more deprived neighborhoods were disproportionately identified for SEND services, but their analysis by need type revealed a social gradient: low‑income students were more frequently assigned speech‑language, social‑emotional needs, or moderate learning difficulty (i.e., moderate intellectual disability) labels, whereas their peers in affluent areas were more often identified with various forms of SpLD or autism. Campbell (2023) argues that these divergent identification patterns suggest that the assessment pathway for SpLD, one that is costlier and often privately commissioned, may be scarcer in England’s most deprived localities.

Other researchers have also shown evidence that SpLD-related diagnoses may not be wholly related to students’ academic proficiency. In a UK study, Knight & Crick (2024) found that children of parents who are in professional or managerial roles were more than twice as likely to be diagnosed with dyslexia compared to children of parents who worked in lower-paid occupations. Odegard etal. (2020) observed that SpLD among students in affluent US schools tend to be more conspicuous and, as a result, their problems appeared to be ‘‘unexpected,’’ leading teachers and parents to frame these difficulties as specific disorders rather than general low attainment, and thereby increasing the probability of an SpLD identification.

## School-Level Factors

Beyond individual characteristics, the sociodemographic composition of schools has been shown to exert an independent influence on the identification of SEND. In England, Lindsay and Strand (2016) examined predictors of speech, language and communication needs (SLCN) and found that students attending primary schools in the highest quintile of free school meal eligibility had half the odds of being identified with SLCN compared with peers in the lowest quintile. They also observed that higher proportions of students from minority ethnic backgrounds or with EAL status were associated with systematically lower rates of SLCN identification. More recently, Hutchinson et al. (2025) demonstrated that school quality, as captured by school inspection ratings, also shaped identification patterns. They observed that children in primary schools rated “inadequate” were around three times more likely to be placed on SEND support compared with peers in schools rated “outstanding.” However, none of the previous studies in England has directly examined how school-level factors shape the identification of SpLD specifically.

Evidence from the United States provides a more mixed picture. Sullivan and Bal (2013), analyzing data from a large urban district, found that student-level sociodemographic factors, particularly poverty, gender, and race, were significant predictors of SpLD identification. In contrast, most school-level compositional variables, such as the proportion of students from minority or low-income backgrounds, had negligible effects once individual characteristics were controlled for. Their findings suggest that, at least in some U.S. contexts, school effects are limited compared to the influence of individual-level disadvantage. Thus, prior research shows that both student characteristics and features of the schools they attend could shape patterns of SEND identification, yet little is known about how these dynamics operate for SpLD specifically in England.

## Current Study

The purpose of this study is to examine factors associated with the identification of SpLD among Year 6 (Equivalent to Grade 5 in the United States) students. By analyzing data from the NPD, the study aims to understand how individual academic performance, school-level factors, and demographic characteristics such as gender, EAL status, and socio-economic status are associated with disparities in SpLD identification. This understanding is crucial for ensuring that students who need support are accurately identified and receive appropriate interventions.

## Research Questions

**RQ1**. What is the association between student-level factors, such as their reading and math scores, their gender, EAL status, and student-level economic deprivation, and their likelihood of being identified with SpLD?
**RQ2**. To what extent do school-level factors such as schools’ average reading and math scores, proportion of EALs, school-level economic deprivation and school membership predict the likelihood of a student being identified with SpLD?

## Method

### Participants

We used data from England’s NPD, a detailed administrative dataset managed by the Department for Education that provides a comprehensive repository of state-funded and private school student performance data. We accessed data for Year 6 students from the 2018/19 academic year. Analyses were restricted to Year 6 pupils in the 2018/19 academic year to ensure that attainment outcomes and SEND identification patterns were not confounded by the widespread disruption to assessments, schooling, and referral practices that may have occurred during the COVID-19 pandemic.

The sample included students who had no special educational needs identification and their peers who were identified with SpLD (see Table 1 for demographic information). We excluded schools that were denoted as special schools and schools that had fewer than five students in their Year 6 class, as these are usually not mainstream schools but special schools or pupil referral units, which are a form of alternate provision settings for students who cannot attend mainstream schools. This reduced the overall sample from 16,192 to 14,887 schools.

**Table 1.** Demographic Information for Study on Disparities in Identification of Specific Learning Difficulties (SpLD) in England.

	Typical students		Students with SpLD	
Characteristic	*n*	%	*n*	%
Gender				
Female	275,911	52.19	6,880	42.27
Male	252,840	47.81	9,396	57.73
English as an Additional Language Learner				
Yes	116,226	22.07	1,525	9.37
No	410,175	77.91	14,742	90.63
Mobile Student				
Yes	39,270	7.43	1,014	6.24
No	489,481	92.57	15,262	93.76
IDACI				
<10%	164,475	31.28	5,462	33.63
10% to 20%	134,213	25.52	4,239	26.10
>20% to 30%	98,085	18.65	2,779	17.11
>30% to 40%	71,804	13.65	2,006	12.35
>40% to 50%	41,567	7.90	1,200	7.39
>50%	15,629	2.97	551	3.39

*Note*. IDACI = Income Deprivation Affecting Children Index. The percentage value reflects the share of children in a specified geographic area affected by income deprivation. For example, an IDACI score of 25% indicates that one in four children in the area is living in an income-deprived family. Higher percentages indicate greater levels of income deprivation among children.

### Variables of Interest

#### Dependent Variable: SpLD Identification

In England, schools must record each student’s primary type of SEND from 12 categories, which include identification labels such as SpLD, speech, language and communication needs, autism spectrum disorder, and sensory and/or physical impairments. According to the SEND Code of Practice (Department for Education, 2015), SpLD covers conditions such as dyslexia, dyscalculia, and dyspraxia. For this study, the dependent variable was a binary indicator of whether a student’s primary recorded need was SpLD or whether they had no SEND identification. Nationally, SpLD accounts for approximately 2.0% of students, compared with about 17% of students identified with any form of SEND.

#### Assessment Scores

Year 6 students in schools across England are annually administered standardized reading and math assessments. The reading assessments assess students’ reading comprehension skills; the math assessments assess students’ math fluency together with computational, problem-solving and reasoning skills. Assessment scores are converted to standard scores from 80 to 120 with a standard deviation of 10 (see Standards and Testing Agency, 2016). The Office for Qualifications and Examinations Regulation reports the reliability of these standardized tests as 0.96 for Mathematics and 0.94 for English (Standards and Testing Agency, 2024).

For the analysis, we aggregated individual student scores within each school to calculate each school’s average Year 6 reading and math scores. To prepare the data for multilevel modeling and to facilitate the interpretation of the results, we scaled both the student-level and school-level scores so that their means were 0, and their standard deviations were 1. These z-scored variables were then used as predictors in the multilevel logistic regression analyses to examine their association with the likelihood of a student being identified as having SpLD or not.

#### Demographic Factors

In addition to student and school-level reading and math scores, we included three key demographic variables in our analysis to control for their potential influence on the likelihood of a student being identified as having SpLD.

##### Gender

Including gender allowed us to examine whether there are gender-based differences in SpLD identification when accounting for students’ academic scores, student demographic information, and school context.

##### English as an Additional Language Status (EAL)

In the National Pupil Database, students who are exposed to a language other than English at home are categorized as EALs. Students’ EAL status can significantly impact academic performance, especially in reading (Daniel, 2025), and may influence the identification of SpLD. We also calculated the proportion of EAL students in each school. While ethnicity data were not available for this study, the EAL flag represents the best available proxy for students’ cultural and language background, though it should be noted that, in England, it primarily captures language exposure and only partially overlaps with ethnic minority status (Lindorff et al., 2025).

##### Income Deprivation Affecting Children Index (IDACI) Score

Socioeconomic disadvantage was operationalized using the IDACI, as pupil-level eligibility for free and reduced-priced meals was not available within the approved data access agreement, and IDACI provided a consistent, area-based proxy for deprivation across the full sample. The IDACI score is a measure of socio-economic deprivation representing the proportion of children under the age of 16 in a given area who live in income-deprived households. It is derived from the National Census data. Note that in the English context, IDACI scores are linked to small geographic areas defined by postcodes, which are typically more granular than U.S. zip codes, often capturing individual streets or parts of streets rather than larger neighborhoods (for further information on the IDACI, see Ministry of Housing, Communities, and Local Government, 2019)

The IDACI score ranges from 0 to 1, with higher scores indicating greater levels of deprivation. The IDACI variable was highly skewed (median = 0.17), and so, consistent with previous research (Strand & Lindorff, 2018), we applied a normal score transformation to produce a variable with a mean of 0 and a standard deviation of 1; scores above 0 indicate greater-than-average deprivation, and scores below 0 indicate less-than-average deprivation, relative to the population average. We also created a school-level average IDACI score, based on the individual-level transformed values, to control for deprivation at the school level.

##### Mobile Students

The National Pupil Database includes a binary predictor indicating whether a student was mobile, defined as having joined their current school during Year 5 or Year 6. This measure captures transitions that may interrupt continuity of instruction and assessment processes. Given prior evidence linking student mobility to differences in academic outcomes (Strand, 1999; Strand et al., 2015), this variable was examined as a potential predictor of SpLD identification.

### Analytical Method

To examine the factors associated with the identification of SpLD among Year 6 students in England, we employed multilevel logistic regression models using the *glmer* function from the lme4 package in R (Bates et al., 2015). To ensure the accuracy and completeness of our analysis, we restricted the multilevel models to schools with complete data for all predictors included in the analysis. This decision was expected to minimize bias due to incomplete cases and dropped <10 schools in our dataset. We also tested a binary indicator of school sector (state-funded vs. private). This variable showed a negligible association with SpLD identification and did not alter the effects of other predictors, so it was excluded from the final models. Accordingly, results are presented for the combined sample of state-funded and private schools.

We analyzed a series of multilevel logistic regression models to assess the association between student-level and school-level factors and the likelihood of a student being identified with SpLD. The dependent variable was binary, indicating whether a student was identified with SpLD (1) or not (0). Given that the outcome (SpLD identification) was measured at the pupil level and pupils were nested within schools, we fitted a series of multilevel logistic regression models with a random intercept for schools. We first estimated an unconditional (empty) model including only a school-level random intercept to quantify the extent of between-school variation in baseline SpLD identification rates. We then introduced pupil-level predictors (assessment score, mobile indicator, EAL status, gender, and IDACI), followed by models that additionally incorporated school-level contextual variables (school-average assessment score, school-level proportion EAL, and school-average IDACI). Random slopes were not included because the primary aim was to estimate average population-level associations rather than model cross-school variation in effects. The null model served as a baseline to estimate the amount of variance in SpLD identification attributable to differences between schools. This model included only a random intercept for schools. Next, we developed separate models for reading and math to examine the effects of individual student academic scores (scaled), student-level factors, school average academic scores (scaled), and school-level factors on SpLD identification. We assessed model fit using Marginal *R*^2^ (variance explained by fixed effects) and Conditional *R*^2^ (variance explained by both fixed and random effects). These measures provide information about the explanatory power of multilevel models by distinguishing variance attributable to fixed effects from that attributable to school-level clustering.

We reported the results of our multilevel logistic regression analyses as odds ratios (ORs) to facilitate interpretation. An odds ratio represents the ratio of the odds of an event occurring to the odds of it not occurring, given a one-unit increase in the predictor variable. In the context of our study, the OR indicates how changes in student-level and school-level factors affect the likelihood of a student being identified with SpLD. An OR greater than 1 suggests that the predictor is associated with increased odds of SpLD identification, whereas an OR less than 1 indicates decreased odds. An OR of 1 implies no meaningful change in the odds of SpLD identification associated with the predictor (Szumilas, 2010).

It is important to note that the NPD encompasses the population of Year 6 students in schools in England rather than a sample. Consequently, conventional null hypothesis significance testing, which is typically used to infer population parameters from sample data, is not appropriate in this context. In line with critiques of significance testing in population-level datasets (Gorard, 2016), we therefore do not report *p*-values or confidence intervals. Instead, we focus on the magnitude and direction of associations, as indicated by odds ratios, and on their substantive and practical significance.

## Results

Table 1 presents the sample’s demographic information, and Table 2 presents the sample’s average reading and math scores. Table 3 summarizes the ORs for the empty model, base models, and full models for both reading and math scores. We first assessed the magnitude of clustering. Intraclass correlation coefficients (ICCs) indicated that 26% of the variability in identification was between schools in the empty model; this rose to 29% to 34% after adding student- and school-level predictors. We then calculated the Median Odds Ratio (MOR), which is the median multiplicative difference in odds between two otherwise identical students attending two randomly selected schools, to express this heterogeneity on the odds scale.

**Table 2.** Descriptive Statistics for Study on Disparities in Identification of Specific Learning Difficulties (SpLD) in England.

	Reading			Math		
Demographic variable	*n*	Mean	*SD*	*n*	Mean	*SD*
School Grand Mean	15,621	104.51	2.76	15,621	106.09	2.46
Student Sample Mean	539,287	105.49	7.53	539,931	106.10	6.45
Students with No SEND	524,073	105.73	7.38	524,501	106.32	6.28
Students with SpLD	15,214	97.24	8.58	15,430	98.30	7.72
Male Students	259,019	104.59	7.53	259,495	106.69	6.48
Female Students	280,268	106.30	7.47	280,436	105.53	6.41
EAL Students	114,826	104.64	7.98	115,451	106.84	6.70
Non-EAL Students	422,958	105.72	7.39	422,891	105.90	6.38
Mobile Student—No	502,442	105.67	7.42	502,511	106.24	6.37
Mobile Student—Yes	36,845	102.92	8.71	37,420	104.00	7.35
Student IDACI Score						
<10% (least deprived)	169,249	106.94	7.19	169,290	107.09	6.16
11-20%	137,386	105.63	7.47	137,538	106.11	6.44
21-30%	99,687	104.74	7.58	99,825	105.65	6.55
31-40%	72,757	104.22	7.63	72,886	105.32	6.60
41-50%	42,106	103.97	7.68	42,180	105.05	6.63
>50% (most deprived)	15,941	103.54	7.64	15,968	104.71	6.56

*Note*. SEND = Special Educational Needs and Disabilities; SpLD = Specific learning difficulty; EAL = English as an additional language learner; IDACI = Income Deprivation Affecting Children Index.

**Table 3.** Probability of Specific Learning Disabilities Identification.

	Reading models			Math models		
Predictors	Empty model	Base model	Full model	Empty model	Base model	Full model
	Odds ratio (*SE*)	Odds ratio (*SE*)	Odds ratio (*SE*)	Odds ratio (*SE*)	Odds ratio (*SE*)	Odds ratio (*SE*)
**Fixed Effects**						
Intercept	0.02 (0.00)	0.01 (0.00)	0.01 (0.00)	0.02 (0.00)	0.01 (0.00)	0.01 (0.00)
*Student-level factors*						
Student Score		0.28 (0.00)	0.27 (0.00)		0.28 (0.00)	0.26 (0.00)
EAL - Yes		0.25 (0.01)	0.24 (0.01)		0.38 (0.01)	0.37 (0.01)
Gender – Male		1.18 (0.02)	1.19 (0.02)		1.98 (0.04)	2.02 (0.04)
IDACI Score		0.88 (0.01)	0.97 (0.01)		0.87 (0.01)	0.96 (0.01)
Mobile Student – Yes		0.63 (0.02)	0.61 (0.02)		0.60 (0.02)	0.64 (0.02)
*School-level factors*						
Avg School Score			1.57 (0.03)			1.51 (0.02)
School EAL %			1.44 (0.11)			0.84 (0.07)
Avg School IDACI			0.92 (0.03)			0.87 (0.02)
**Random Effects**						
Level-1 Residual	3.29	3.29	3.29	3.29	3.29	3.29
Level-2 Intercept	1.17	1.62	1.37	1.17	1.73	1.48
ICC	0.26	0.33	0.29	0.26	0.34	0.31
*N* (schools)	14,886	14,886	14,886	14,882	14,882	14,882
Observations	537,787	537,787	537,787	53,8345	538,345	538,345
Marginal *R*^2^/Conditional *R*^2^	0.00/0.26	0.27/0.51	0.28/0.49	0.00/0.26	0.27/0.52	0.28/0.50

*Note*. EAL = English as an additional language learner; IDACI = Income Deprivation Affecting Children converted to normal distribution. Student and school average reading and math scores were z-transformed.

For the math models, the MORs were 2.81 (empty model), 3.21 (base model), and 3.07 (full model), suggesting that for the median pair of schools, the odds of a student being identified are nearly three times higher in one school than the other, even after adjusting for measured student and school characteristics. The increase in ICCs and MORs from the empty to the base model is consistent with multilevel logistic modeling, where the level-1 residual variance is fixed and reducing within-school variability can make between-school differences more apparent (Leyland & Groenewegen, 2020); the negligible change after adding school-level covariates indicated that the observed school measures explained little of the residual clustering.

Reading models showed a similar pattern, with MORs of 2.81, 3.36, and 3.06 and ICCs of 0.26, 0.29, and 0.33 in the empty, base, and full models, respectively. A caterpillar plot of the random intercepts for 50 randomly selected schools is provided in Appendix A, which visually illustrates the wide range of school-specific effects and highlights the significant heterogeneity in the odds of identification across schools. However, a small proportion of schools are observed above the mean, indicating that a subset of schools drives the between-school variation, consistent with the observed right skew in the caterpillar plot.

### Student-Level Predictor Models

The student-level predictor models included students’ (scaled) reading or math scores and their sociodemographic factors. As would be expected, the odds ratio indicates that as students’ reading or math scores increase, their likelihood of being identified for SpLD decreases. More specifically, one standard deviation above the mean on students’ reading scores was associated with lower odds of SpLD identification (OR = 0.28), and each additional standard deviation above the mean on their math scores was associated with lower odds of SpLD identification (OR = 0.28), while controlling for student-level sociodemographic factors.

Students with EAL status had a significantly lower likelihood of being identified with SpLD compared to their non-EAL peers. Controlling for academic scores and other sociodemographic factors, the odds of SpLD identification for EAL students were lower in the reading model (OR = 0.25) and in the math model (OR = 0.38). This substantial decrease indicates potential under-identification of SpLD among students identified as EALs. Similarly, when accounting for student-level economic deprivation, a one-standard-deviation increase in the IDACI score (indicating greater deprivation) was associated with lower odds of SpLD identification (reading model OR = 0.88; math model OR = 0.87).

Year 6 male students were more likely to be identified for SpLD than female students (OR = 1.18) while controlling for students’ reading scores and other sociodemographic factors. Notably, the effect of gender was more pronounced in the math model. The odds of identification were almost twice as high for boys compared to girls (OR = 1.98), suggesting a greater gender disparity in the model controlling for their math test scores.

Finally, students who joined their current school in Year 5 or Year 6 (i.e., mobile students) were at substantially lower odds of SpLD identification, with odds ratios of 0.63 in the reading model and 0.60 in the mathematics model. This pattern suggests that school transitions may limit opportunities for identification, potentially due to reduced teacher familiarity with the student’s learning history or fewer cumulative observations of their academic performance.

### Student- and School-Level Predictor Models

Student-level predictors remained mostly stable after controlling for school-level predictors. The schools’ average reading score (scaled) had a meaningful impact on the students’ SpLD identification (OR = 1.57). Similarly, a one-standard-deviation increase in the school’s average math score (scaled) was associated with an increase in the odds of SpLD identification (OR = 1.51). Findings suggest that the school-level mean achievement exerts a sizeable contextual effect: students are more likely to be labeled SpLD in high-achieving schools, indicating that identification is partly relative to peer performance rather than absolute skill levels.

Additionally, a one-standard-deviation increase in the school-level average IDACI score was associated with a decrease in the odds of SpLD identification in the reading model (OR = 0.92) and in the math model (OR = 0.87), indicating that higher levels of school-level deprivation are linked to a lower likelihood of SpLD identification. Finally, the proportion of EAL students in a school was positively associated with SpLD identification in the reading model (OR = 1.44) but showed a negative association in the mathematics model (OR = 0.87). This suggested that the influence of school-level language diversity on identification patterns may be subject-specific.

## Discussion

This study used a large, national dataset to examine how student-level academic performance, sociodemographic characteristics, and school-level factors are associated with the likelihood of Year 6 students in England being identified with SpLD. As expected, lower reading and mathematics scores were strongly associated with higher odds of identification, aligning with the view that academic underperformance is a central factor in SpLD identification (Fletcher et al., 2019; Hajovsky et al., 2025). However, by modeling individual and contextual factors simultaneously, the findings also demonstrate that identification is not solely a function of a student’s attainment profile but is shaped by demographic characteristics and the institutional context in which they are educated.

This paper began with a thought experiment: To what extent is it appropriate and acceptable for a given child, with a given profile of skills and achievement, to be considered to have an SpLD if they attend School A rather than School B? The present findings suggest that such scenarios are not purely hypothetical. We found that even when students share similar levels of academic performance, their likelihood of being identified with SpLD varied systematically according to factors beyond their reading and math skills, including their language background, socioeconomic circumstances, gender, mobility status, and their school-level composition.

The analyses suggest that a child with an EAL background, even with the same reading or mathematics score as a non-EAL peer, is substantially less likely to be so identified. These patterns suggest that SpLD identification is shaped not only by individual need but also by the contextual lens through which schools consciously or subconsciously interpret “unexpected underachievement.” Gender differences were also pronounced, particularly in the mathematics models, where boys had about twice the odds of being identified compared to girls with similar math attainment. This finding supports prior research suggesting that the greater propensity for boys to present problematic externalizing behaviors may trigger more male referrals for assessment (Shaywitz, 1996), while girls’ difficulties, especially when accompanied by internalizing behaviors, may remain less visible in classroom settings, and even if identified, may not be prioritized for referral (Pennington, 2008). Thus, two students with the same mathematics score could be perceived differently depending not only on their achievement profile alone, but potentially also on gendered expectations and behavioral presentations that possibly influence identification and referral decisions. Such patterns raise concerns about the risk of overidentifying boys and underidentifying girls, with corresponding implications for access to additional support.

Socioeconomic deprivation, measured at both the individual and school level using the IDACI score, was consistently associated with lower odds of SpLD identification in our models. This finding supports previous evidence that pathways to identification may be more accessible in affluent communities, where resources for assessment, often requiring private assessments, are more readily available (Campbell, 2023; Knight & Crick, 2024). Our findings suggest that, although “unexpectedness” is not an explicit criterion for SpLD identification in the United Kingdom (as it has historically been in the United States), the academic norms and resources of the surrounding context may nonetheless shape perceptions of underachievement in ways that influence identification, rather than it being based purely on an objective measure of academic performance.

Mobility status emerged as a unique indicator in the context of SpLD identification. This finding aligns with Hutchinson et al. (2025), who reported that moving schools was associated with lower odds of identification for any student with SEND. However, we are not aware of any prior research that has specifically examined mobility status as a predictor of SpLD identification while controlling for students’ academic achievement, making this a novel contribution of the present study. Our results indicate that students who joined their current school in Year 5 or Year 6 were markedly less likely to be identified with SpLD, even after accounting for attainment and other relevant factors. This suggests that mobility may disrupt the accumulation of observational data, teacher familiarity with a student’s learning history, and continuity in the assessment process. Although Government guidelines require all student records to be transferred to their new school within a 15-day window (Department for Education, n.d.), delays or gaps in the quality and completeness of these records may still hinder timely identification.

A further striking finding concerned the role of the school’s overall academic context. Specifically, students attending schools with higher average reading and mathematics scores were substantially more likely to be identified with a SpLD, even when their individual attainment was held constant. This finding indicates that an individual’s outcomes are evaluated relative to the performance of their peers rather than against an absolute benchmark. In the present study, this manifests as an increased likelihood of identification in high-achieving schools, suggesting that “unexpected underachievement” is partly constructed through local academic norms. The implication is that SpLD identification does not occur in a vacuum: students with identical test scores may be perceived differently depending on the achievement profile of their school, reinforcing the view that contextual factors shape pathways to SpLD identification.

### Implications

These findings hold important implications for both practice and policy. To address potential contextual biases stemming from differing school practices or standardized diagnostic criteria, there is a need for guidelines for SpLD identification to be established at a national level. Similar calls for greater standardization have been made in other contexts (e.g., Fletcher & Miciak, 2024; Kavale et al., 2009; Pérez, 2020), suggesting that the challenge is not unique to England but reflects wider international concern. Such frameworks might help reduce unwarranted variability and promote more equitable identification practices and access to services across diverse educational settings. A starting point in England would be the government guidelines specifying what constitutes SpLD. Although the SEND Code of Practice in England recognizes SpLD as a disability, it fails to provide a definition or guidelines on how a child may be identified. However, it is recognized that this may be particularly challenging as recent studies have critiqued that “*all current methods of SpLD identification are insufficient to adequately identify individuals with SpLD*” (Hajovsky et al., 2025, p. 10).

Furthermore, our results underscore a critical need for professional development targeting teacher and parental awareness in order to counter referral biases. Gender disparities might be addressed through training and guidelines that sensitizes educators and caregivers to subtle behavioral and performance indicators across genders. Similarly, improved training on differentiating linguistic development, alongside culturally responsive assessment tools, could mitigate under-identification of EAL students (Fagan & Herrera, 2022).

Targeted investment in disadvantaged schools to improve assessment, identification and support is warranted. Ensuring sufficient resources and strengthening school-home-community partnerships could enhance early identification and effective support strategies for students who are generally considered at higher risk of SpLD (Darling-Hammond, 2019). Moreover, past studies have reported that culturally and linguistically diverse families generally report several barriers to accessing support services for their children with additional needs (Akbar & Woods, 2020; Rossetti et al., 2021). Thus, fostering parental advocacy and engagement in communities with historically lower identification rates might encourage more equitable SpLD identification and intervention.

### Limitations

Several limitations should inform cautious interpretation of these results. The cross-sectional nature of our data limits causal inference. Longitudinal studies tracking identification patterns and academic trajectories over time could provide insights to ascertain whether earlier identification and subsequent intervention affected Year 6 assessment outcomes or whether delayed identification exacerbated existing learning difficulties.

Additionally, our analysis relied upon the NPD’s categorization of EAL status, without accounting for students’ English proficiency, first language literacy, or length of exposure to English-medium education. An additional limitation is the lack of access to ethnic or racial background data, which prevented controlling for these factors in our models. Previous research highlights significant disproportionalities in special needs identification linked to students’ race or ethnicity, suggesting biases or systemic inequities in assessment and referral practices (e.g., Shifrer et al., 2011; Strand & Lindorff, 2018; Strand & Lindorff, 2021; Sullivan & Bal, 2013). Without accounting for ethnicity or race, it is unclear whether the observed patterns in SpLD identification, such as under-identification among EAL students or variations across socioeconomic contexts, may be partially explained by racial or ethnic factors.

The use of IDACI as a proxy for parental income represents another limitation, as this may obscure considerable variability in household socioeconomic status within deprived areas. Employing individual-level socioeconomic measures would provide more accurate insights into the specific effects of socioeconomic factors. Again, in our dataset, we did not have access to students’ free and reduced-priced meal data, which could have provided a more direct indicator of individual-level economic disadvantage and allowed for a finer-grained analysis of how household socioeconomic status relates to SpLD identification.

Another limitation of our study is the inability to measure the full range of school-level practices that may influence SpLD identification. Despite controlling for several student- and school-level factors, the multilevel models showed substantial and persistent between-school heterogeneity, indicating that a large proportion of the variability in SpLD identification remains unexplained by the variables in our model. These findings suggest that the substantial school-level effects we observed likely reflect unmeasured influences, such as school-specific policies, referral procedures, teacher training, or parental advocacy. As these variables were not available in the NPD, their influence on identification practices could not be captured. Investigating these unmeasured school-level processes and practices is therefore an important area for future research. Finally, generalizability is constrained to the population of Year 6 students in England. Extending analysis across other educational contexts could strengthen the external validity of our findings.

## Figures and Tables

**Figure A1.**
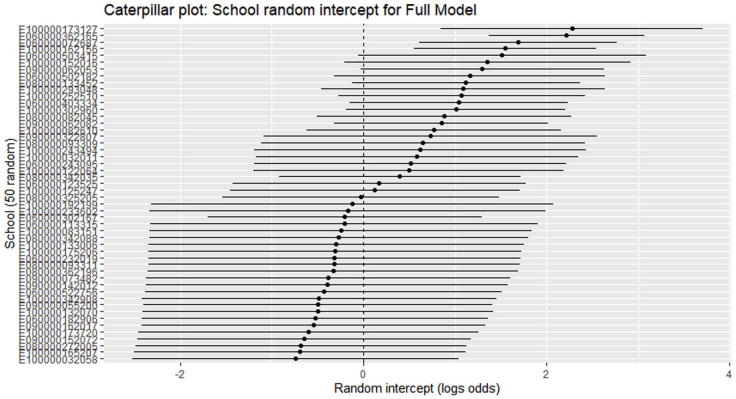
Caterpillar Plot of School Random Intercept (Log-Odds) from the Full Logistic Regression Reading Model: Random Subset of 50 Schools.

## Appendix A

### Random Effects of the Full Multilevel Logistic Regression Reading Model

Figure A1 presents a caterpillar plot of the random intercepts for 50 randomly selected schools from the full multilevel logistic regression reading model. This plot visually illustrates the between-school variability in the log-odds of students being identified with a Specific Learning Difficulty (SpLD), after accounting for all student- and school-level predictors. Each horizontal line represents a school, with the central black dot indicating the school’s estimated random intercept. The line extending from the dot represents the 95% confidence interval for that estimate. The dashed vertical line at zero represents the average random intercept across all schools. Schools with confidence intervals that do not cross the zero line have an odds of identification that is significantly different from the average school in the sample. As shown in the figure, there is a range of random intercepts, with many schools having confidence intervals that do not overlap with the average, providing a visual confirmation of the substantial and significant heterogeneity in SpLD identification practices across schools that was quantified by the models’ Intraclass Correlation Coefficient and Median Odds Ratio.

## Appendix B

**Table B1.** Correlations Between Continuous Variables.

Variable	Student Reading Score	Average School Reading Score	Student Math Score	Average School Math Score	IDACI
Student reading score	1				
Average School Reading Score	0.33	1			
Student Math Score	0.65	0.29	1		
Average School Math Score	0.28	0.76	0.38	1	
IDACI	-0.14	-0.32	-0.11	-0.16	1

*Note.* IDACI = Income Deprivation Affecting Children Index.

All *p-values* <.01.
